# CT Perfusion Imaging as an Early Biomarker of Differential Response to Stereotactic Radiosurgery in C6 Rat Gliomas

**DOI:** 10.1371/journal.pone.0109781

**Published:** 2014-10-17

**Authors:** Timothy Pok Chi Yeung, Maher Kurdi, Yong Wang, Baraa Al-Khazraji, Laura Morrison, Lisa Hoffman, Dwayne Jackson, Cathie Crukley, Ting-Yim Lee, Glenn Bauman, Slav Yartsev

**Affiliations:** 1 Department of Medical Biophysics, Western University, London, Ontario, Canada; 2 Robarts Research Institute, Western University, London, Ontario, Canada; 3 Department of Pathology, Western University, London, Ontario, Canada; 4 Department of Pathology, King Abdulaziz University, Jeddah, Makkah, Saudi Arabia; 5 Department of Anatomy and Cell Biology, Western University, London, Ontario, Canada; 6 Lawson Imaging, Lawson Health Research Institute, London, Ontario, Canada; 7 Department of Medical Imaging, Western University, London, Ontario, Canada; 8 Department of Oncology, Western University, London, Ontario, Canada; 9 London Regional Cancer Program, London, Ontario, Canada; University of Nebraska Medical Center, United States of America

## Abstract

**Background:**

The therapeutic efficacy of stereotactic radiosurgery for glioblastoma is not well understood, and there needs to be an effective biomarker to identify patients who might benefit from this treatment. This study investigated the efficacy of computed tomography (CT) perfusion imaging as an early imaging biomarker of response to stereotactic radiosurgery in a malignant rat glioma model.

**Methods:**

Rats with orthotopic C6 glioma tumors received either mock irradiation (controls, *N* = 8) or stereotactic radiosurgery (*N* = 25, 12 Gy in one fraction) delivered by Helical Tomotherapy. Twelve irradiated animals were sacrificed four days after stereotactic radiosurgery to assess acute CT perfusion and histological changes, and 13 irradiated animals were used to study survival. Irradiated animals with survival >15 days were designated as responders while those with survival ≤15 days were non-responders. Longitudinal CT perfusion imaging was performed at baseline and regularly for eight weeks post-baseline.

**Results:**

Early signs of radiation-induced injury were observed on histology. There was an overall survival benefit following stereotactic radiosurgery when compared to the controls (log-rank *P*<0.04). Responders to stereotactic radiosurgery showed lower relative blood volume (rBV), and permeability-surface area (PS) product on day 7 post-stereotactic radiosurgery when compared to controls and non-responders (*P*<0.05). rBV and PS on day 7 showed correlations with overall survival (*P*<0.05), and were predictive of survival with 92% accuracy.

**Conclusions:**

Response to stereotactic radiosurgery was heterogeneous, and early selection of responders and non-responders was possible using CT perfusion imaging. Validation of CT perfusion indices for response assessment is necessary before clinical implementation.

## Introduction

Patients with glioblastoma multiforme, the most common form of adult brain tumors, have a median survival of approximately 12–15 months [1]. Radiotherapy has played an important role in prolonging the survival of these patients since the 1970s [2]. Magnetic resonance (MR) and computed tomography (CT) are currently the standard imaging modalities for assessing treatment response. Tumor progression is usually detected by increased contrast-enhancement on gadolinium-enhanced T1-weighted MR images, and T2-weighted or fluid-attenuated inversion recovery MR images [3]. Gadolinium-enhanced T1-weighed MR is a snapshot of contrast-enhancement in brain tissue and tumor after contrast injection. However, assessment of tumor size based on contrast enhancement after radiotherapy is not a specific tool to predict treatment failure as changes in contrast enhancement can occur in pathophysiological processes that disrupt the blood-brain barrier. For instance, radiation-induced injury (e.g. radiation-induced necrosis and pseudoprogression) can mimic the appearance of tumor progression on post-gadolinium T1-weighted MR [3].

Due to the poor survival rates, timely and accurate assessment of response to radiation is important as treatment can be altered if a non-responder to radiation can be identified early enough. In preclinical studies, radiotherapy is usually delivered using a technique called stereotactic radiosurgery (SRS). It is delivered in one or a few large dose fractions of 8–30 Gy and results in tumor growth delay and survival benefit [4]–[7]. Radiotherapy, particularly SRS, can result in vascular damage in the irradiated area [4], [8]–[10], and radiosensitivity of tumor vasculature can augment response to SRS [11].

Hypofractionation radiotherapy that also use a large dose per fraction to treat patients with malignant gliomas is also an attractive treatment strategy. Recently, clinical studies have investigated the use of hypofractionated intensity-modulated radiotherapy to treat these patients [12]–[14]. Hypofractionation has some advantages over conventional fractionation. Hypofractionation is expected to increase the biological effect of radiation due to increased cell damage resulting from a higher dose per fraction [15]. It also reduces the effect of tumor repopulation by reducing treatment time [16].

Since high-dose per fraction radiotherapy (e.g. SRS and hypofractionated radiotherapy) can result in vascular damage, imaging of tumor perfusion could be a promising biomarker of response to this type of radiation treatment. The C6 rat glioma is a widely used experimental model for the study of malignant glioma [17], including the effects of SRS [6], [7], [9], [10]. Tumor microcirculation can be noninvasively evaluated by CT or MR perfusion imaging techniques. Both perfusion techniques acquire repeated CT or MR images in succession after contrast injection; thus, the wash-in and wash-out of the contrast in tissue can be modeled with tracer kinetic analysis. CT perfusion imaging is a method that allows quantitative mapping of blood flow (BF), blood volume (BV), and permeability-surface area product (PS) [18], [19]. CT scanners and iodinated contrast agents are also widely available, and they are ubiquitous in radiation oncology departments. The purpose of this study was to evaluate vascular changes following SRS using CT perfusion, and determine whether acute vascular changes within one week of SRS is predictive of survival. In addition, early (four days post-SRS) and late (eight weeks post-SRS) histopathologic findings were examined.

## Materials and Methods

This project was approved by the University Council on Animal Care (Project #2010-009) at Western University.

### C6 Glioma Model

Male Wistar rats weighing 300–400 g (*N* = 33) were used in this study (Charles River Canada, age 8 to 10 weeks at surgery). The animals were anaesthetized with 2% isoflurane during all procedures. C6 glioma cells (CCL-107, American Type Culture Collection, Manassas, VA) were cultured in F12k 15% horse serum, 2.5% bovine serum, and 1% penicillin-streptomycin. For the implantation of C6 glioma cells, each animal was secured into a stereotactic surgical frame. After scalp incision at midline and exposing the bregma, a 1 mm diameter burr hole was drilled at 1 mm anterior and 3 mm right of the bregma. A total of 10^6^ C6 glioma cells were slowly injected for 5 minutes at a depth of 3–4 mm from the skull surface, which corresponded to the location of the caudate putamen [20]. The burr hole was sealed with bone wax, and the scalp was closed with sutures.

### Baseline CT Perfusion Imaging

All rats underwent the first CT perfusion scan on an average of 11 days (range, 7–16 days) after implantation of C6 glioma cells to monitor tumor growth and prepare for SRS. The rats were scanned using a clinical CT scanner (Discovery 750 HD, GE Healthcare, Waukesha, WI). A two-phase CT perfusion scan, guided by a prior non-contrast CT scan that identified 16×1.25 mm thick sections to cover the entire brain, was performed for each animal. The brain was scanned with high resolution mode for 32 s at 1.4 s intervals during the first phase and for a period of 165 s at 15 s intervals during the second phase. A bolus of contrast (Isovue, Bracco Diagnostics Inc, Vaughan, Canada, 300 mg iodine/ml, 2.5 mL/kg body weight) was injected into the lateral tail vein at a rate of 0.13 mL/s at 3–4 s after the start of the first phase. The scanning parameters were 80 kVp, 120 mAs, 10 cm field of view, and high-definition bone filter. The visibly distinguishable spatial resolution was 1 line pair per 500 µm measured on a rat-size phantom [21].

CT perfusion images of the same slice were averaged over the whole scan duration using the prototype version of CT perfusion 4D (GE Healthcare) to produce a set of contrast-enhanced images called averaged CT images. The tumor diameter on the averaged CT images was measured immediately after the scan. The rats were assigned randomly to either the control group (*N* = 8) or the SRS group (*N* = 25) once a tumor diameter of 4 mm was reached.

### Stereotactic Radiosurgery

A custom-made plastic stereotactic frame was used to secure the animals for treatment planning. A non-contrast helical CT scan was performed (25 cm field-of-view, 80 kV, 110 mAs, 1.25 mm slice thickness) for treatment planning. CT images were transferred to the Helical Tomotherapy Treatment Planning System (Accuray Inc, Madison, WI, USA). Using the averaged CT images for anatomical guidance, a dose of 12 Gy was prescribed to at least 80% of the contrast-enhanced tumor volume with the brainstem receiving no more than 4 Gy and less than 10% of the normal brain receiving more than 8 Gy. Figure S1 shows an example of a treatment plan.

SRS was delivered using version 4.2 Hi-ART Helical Tomotherapy (Accuray Inc., Sunnyvale, CA). The rats were fixed in the same plastic stereotactic frame. A 3.5 MV CT study was acquired prior to treatment and was co-registered with the planning CT study. Then the couch position was shifted in the lateral, anterior-posterior, and superior-inferior directions to correct translational positioning errors. The rats were repositioned in the stereotactic frame if there were rotational positioning errors. The average SRS delivery time was 6 minutes (6 MV, 825 cGy/min). Rats in the control group were put under anesthesia and secured in the stereotactic frame for 6 minutes to mimic the procedure without radiation.

### Follow-up CT Perfusion Imaging

Rats in the SRS group were further assigned to either 1) acute imaging group (*N* = 12) or 2) the longitudinal imaging group (*N* = 13) (Figure S2). Rats in the acute imaging group were imaged with CT perfusion at 4 days post-SRS, and were euthanized on the same day. Rats in the longitudinal group and control group were imaged for a maximum of 8 weeks post-SRS to monitor tumor changes and survival. Rats were euthanized if they showed any of the following signs and symptoms: weight loss of ≥15% from the heaviest recorded weight, lethargy, refusal to eat, neurologic signs such as one-sided limb weakness.

### Image Analysis

**Figure 1:**
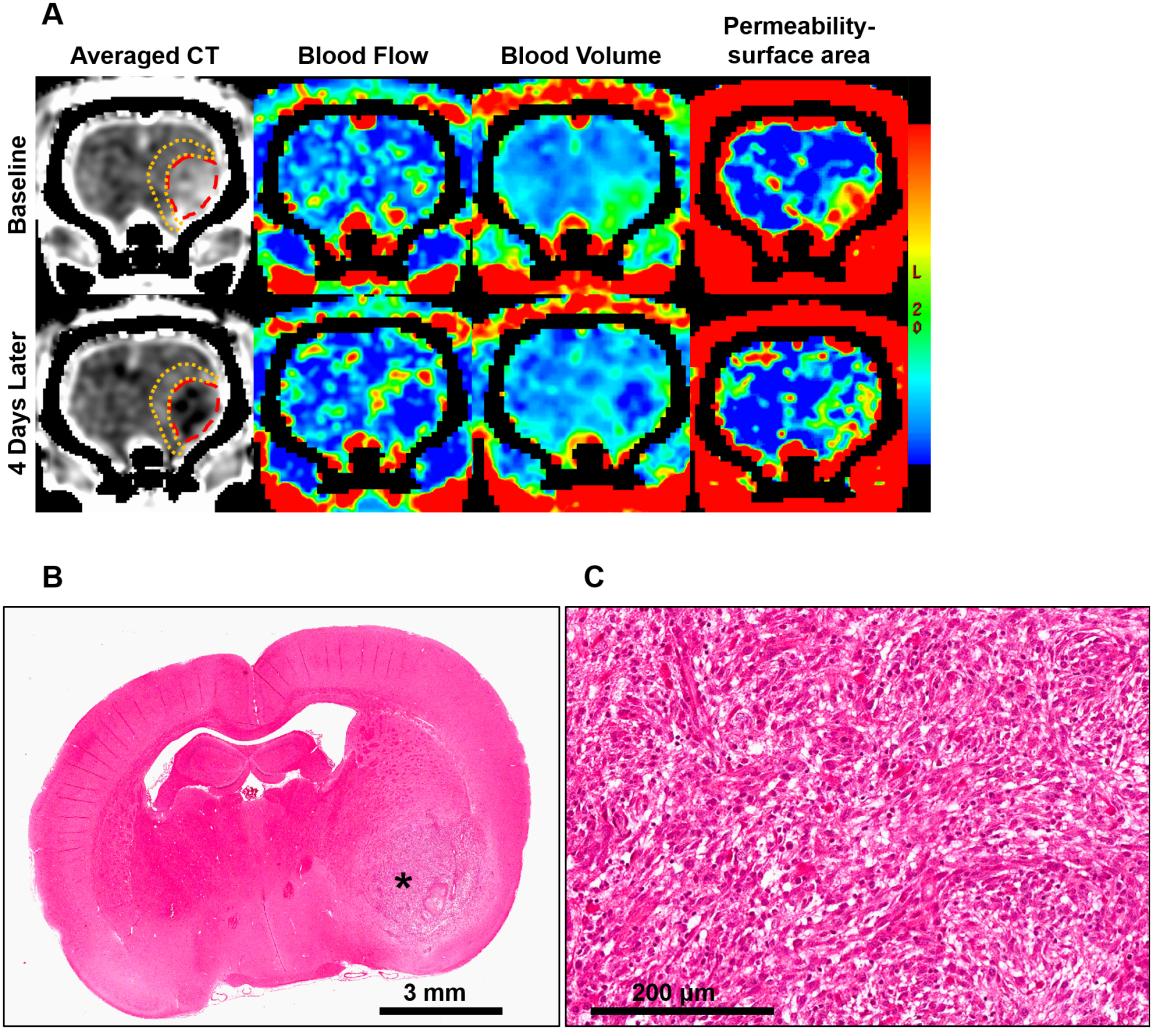
Acute changes after stereotactic radiosurgery (SRS). (A) Acute CT perfusion changes between baseline and the fourth day after SRS. Tumor is outlined in red and the peritumoral region is outlined in orange. For this animal, tumor relative blood flow (rBF) was 1.48 at baseline and it decreased to 0.68 after SRS. Tumor relative blood volume (rBV) decreased from 1.70 to 0.78, while permeability surface-area (PS) decreased from 4.67 to 3.87 ml/min/100g. On the contrary, peritumoral rBF increased from 0.96 to 1.49, rBV increased from 1.06 to 1.52, and PS increased from 2.13 to 3.81 ml/min/100g. (B) Hematoxylin and Eosin (H&E) image of this animal after radiosurgery. Asterisk indicate the location of the tumor. (C) Magnified H&E image of the tumor at the location indicated by the asterisk in (B).

Maps of BF, BV, and PS were generated using the prototype version of CT perfusion 4D (GE Healthcare). The time-attenuation curve (TAC) from the carotid artery was selected as the arterial input. The arterial TAC was deconvolved with tissue TACs measured from 2×2 pixel blocks of CT images using the Johnson-Wilson model to produce maps of BF, BV, and PS [18], [22]. Using the averaged CT images for anatomical guidance, the lesion that was contrast-enhanced before SRS and the same lesion that became non-enhanced after SRS was delineated as the tumor (Figure 1). The region that is 2–3 mm adjacent to the tumor that became contrast-enhanced after SRS was delineated as the peritumoral region. The contralateral normal brain was also delineated. Tumor volume, BF, BV, and PS in the tumor, peritumoral region, and contralateral normal brain were measured. Mean BF and BV values were normalized to the contralateral normal brain to obtain the relative BF (rBF) and relative BV (rBV) values.

### Histopathologic Examination

On the same day as the last CT perfusion scan, the animals were euthanized with an overdose of potassium chloride. The animals were perfusion-fixed with phosphate buffered saline followed by 4% paraformaldehyde. The brains were removed and fixed in 4% paraformaldehyde for 24 hours. The brain specimens were sectioned into 3 mm thick blocks, paraffin-embedded, then sectioned at 5 µm. Hematoxylin and eosin stain (H&E) was applied on each section.

A blinded neuropathology resident (M.K.) examined all H&E slides at different magnifications (20x and 40x) to evaluate histologic signs of radiation damage. The presence of tumor was evaluated as “no”, “yes”, or “diminished due to radiation effect”. “Yes tumor” means the presence of a hypercellular lesion. “Diminished due to radiation effect” means the presence of a circumscribed hypocellular lesion showing features of irradiation. “No tumor” means the absence of a hypercellular lesion and a hypocellular lesion. The diameter of each lesion (control and SRS-treated animals) was measured. Tumor cellularity was defined as 1) hypercellular when less than 30% of the tumor area appeared pale or major tumor was found, and 2) hypocellular when more than 50% of the tumor area appeared pale excluding necrosis. Tumor was graded based on the World Health Organization criteria [23]. Tumor edema was defined as the presence of extensive intracellular cytoplasmic swelling or intercellular spaces between tumor cells. Peritumoral edema was defined as intracellular cytoplasmic swelling or presence of intercellular spaces and reactive gliosis in the peritumoral region. Necrosis was described as 1) pseudopalisading necrosis that indicates high-grade glioma, 2) fibrinoid necrosis, or 3) background necrosis (neither pseudopalisading nor fibrinoid necrosis) that indicate post-radiation effect. Presence of endothelial hyperplasia was recorded as a feature of angiogenesis in high-grade glioma. Increased vascularity and hyalinzed blood vessels were recorded as features of post-radiation effect. Percentage of animals exhibiting these signs was calculated. Mean percent necrosis by area was also calculated.

The brain sections were also stained with a Cy3-conjugated mouse monoclonal anti-α-smooth muscle actin (α-SMA) (1∶500, clone 1A4, C6198, Sigma-Aldrich) to identify mature vessels [24]–[26]. The slides were scanned using an Aperio ScanScope (Leica Biosystems, Vista, CA), and images were captured using the ImageScope software (version 11.2.0.780, Leica Biosystems, Vista, CA). The α-SMA positive vessels were qualitatively classified as intact or fragmented vessels. For each slide, α-SMA positive vessel density was defined as the number of vessels that stained positive for α-SMA in 6 separate fields (20×) in the most vascular region of the tumor and peritumoral region. An average of 14 separates fields in the tumor and 14 separate fields in the peritumoral region were examined for each animal. α-SMA positive vessels were manually counted using the software JMicroVision [27]. Intact and fragmented α-SMA positive vessel densities were measured separately. The percentage of fragmented α-SMA positive vessels was also recorded.

### Statistical Analysis

The Shapiro-Wilk test was used to test the normality of the data. Temporal changes in the acute imaging group were tested by the Wilcoxon signed-rank test. The end point of the longitudinal imaging group was survival at 8 weeks post-SRS. The longitudinal group was also subdivided into those that survived more than 15 days (responder group), and those that survived less than 15 days post-SRS (non-responder group). The omnibus Kruskal-Wallis test was used for comparisons between groups followed by the Mann-Whitney *U* test. The omnibus Friedman test was used for longitudinal comparisons within-group followed by the Wilcoxon signed-rank test. All data were reported as mean ± standard error of the mean (SE). Statistical comparisons were performed for the first 4 time points (i.e. day 14 post-SRS) because all controls died before the 5^th^ time point (i.e. day 21 post-SRS).

Survival of the groups was compared using the log-rank test. Spearman’s rank correlation was used to assess the associations between different imaging parameters (tumor volume, rBF, rBV, PS) and the percentage of fragmented α-SMA positive vessels. PS is the product between microvessel surface area and permeability, and BV has been shown to correlate with microvessel area [28]. Thus, we used the PS:BV ratio as a surrogate of permeability and correlated this parameter with the percentage of fragmented α-SMA positive vessels as well. Correlation between tumor volume measured from averaged CT images and tumor diameter measured from H&E histology was examined.

We investigated whether the imaging parameters correlated with overall survival, and whether these parameters on day 7 post-SRS could predict survival. Each imaging parameter was assessed by applying a threshold that is below the lower 50% confidence interval (CI) boundary of the variations across all treated animals derived from the between-subject variation on day 7 post-SRS. This threshold was used to categorize each animal into two groups (i.e. higher or lower than the threshold). Survivals of the different groups were compared. The sensitivity, specificity, and accuracy of each parameter in predicting survival were assessed. A *P* value≤0.05 was considered statistically significant.

## Results

### Acute Vascular Changes and Histopathologic Features

Irradiated rats received 12 Gy in the tumor and peritumoral region, and different CT perfusion changes were observed in these two regions. After SRS, tumor volume, rBF, and rBV decreased significantly compared to baseline (*P*≤0.05) while peritumoral rBF, rBV, and PS increased significantly compared to baseline (*P*≤0.05) (Table 1). These opposite CT perfusion changes in the tumor and peritumoral regions are illustrated in Figure 1A. For the control animals, these parameters did not change significantly from baseline (*P*>0.05).

**Table 1:** Acute changes (± standard error) in CT perfusion parameters.

		Percent Change (%)			
Regions-of-interest	Treatment	Tumor volume	rBF	rBV	PS
Tumor	SRS	−37.3±13.2* ^,^ ##	−18.2±7.9*	−23.1±7.6*	−9.2±9.7
	Control	95.1±34.7*	−13.1±9.3	−6.4±9.4	7.4±15.1
Peritumoral	SRS	N/A	20.9±7.9* ^,^ #	28.7±7.7** ^,^ #	54.3±11.6**
	Control	N/A	−5.0±5.3	2.0±5.0	13.9±20.2

*Abbreviations*: SRS, stereotactic radiosurgery; rBF, relative blood flow; rBV, relative blood volume; PS, permeability-surface area product.

*Significantly change from baseline at *P*≤0.05 level.

**Significantly change from to baseline at *P*≤0.01 level.

# Percent change significantly different than control animals at *P*≤0.05 level.

## Percent change significantly different than control animals at *P*≤0.01 level.

**Figure 2:**
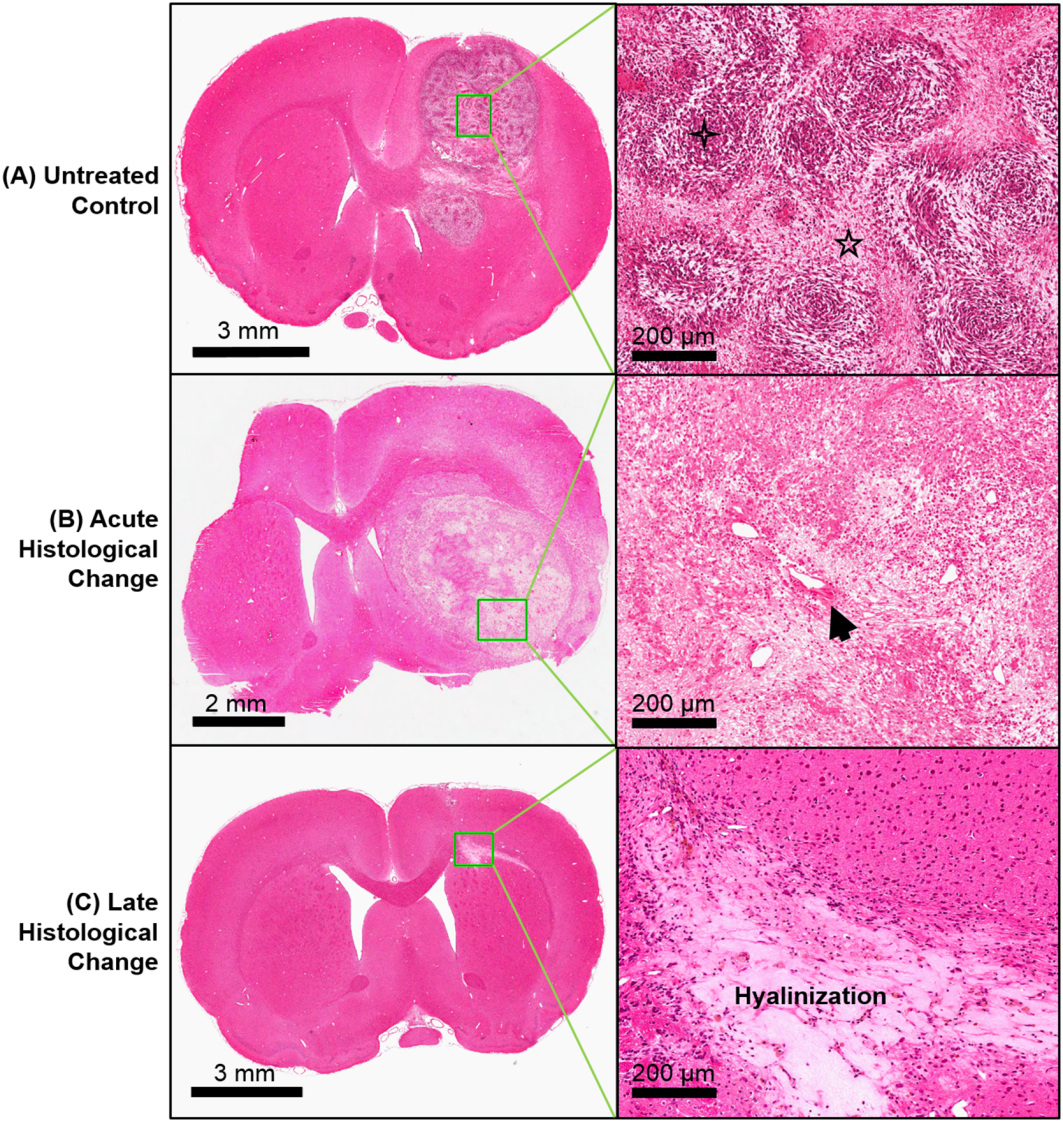
Histological examples of stereotactic radiosurgery (SRS) effects. Representative Hematoxylin & Eosin images of (A) an untreated control, (B) acute histological change at 4 days post-SRS, and (C) late histological change at 59 days post-SRS. Hypercellularity (four point star) and pseudopalisading necrosis (five point star) are classic signs of grade IV glioma, and these were observed in the control animals. Hyalinized blood vessels (arrow) and hypocellularity can be observed shortly after SRS. Regression of tumor and hyalinization of tissue were observed at a later stage after SRS.

H&E histological examination revealed that all tumors were grade IV gliomas. Hypercellularity, mitosis, pseudopalisading necrosis, and endothelial hyperplasia were observed as signs of grade IV glioma in the control animals. For the acute imaging group that received SRS, fibrinoid or background necrosis, increased vascularity, and hyalinized blood vessels were identified. Atypical nuclei, calcification, and gliosis were also observed in some lesions. Examples of H&E images of unirradiated tumor and acute effects of SRS are demonstrated in Figure 2A and 2B, respectively. A summary of histological findings is provided in Table 2.

**Table 2:** Summary of histological finding on Hematoxylin and Eosin (H&E) specimens.

	Presence of Tumor	Tumor Diameter(mm)	Hypo-cellularity	Types ofNecrosis	% AreaNecrosis	Vascularity	Tumor Edema	Peritumoral Edema
Controls (*N* = 8)	88% Yes	7.3±4.0	0%	88% PPN	46±27	75% EH	0%	0%
	0% Diminished			13% FN		25% Increased		
						25% HBV		
Acute Histology (*N* = 12)	75% Yes	5.8±3.0	25%	50% PPN	37±19	58% EH	25%	42%
	25% Diminished			25% FN		58% Increased		
				33% BN		100% HBV		
Responders (*N* = 7)	0% Yes	N/A	N/A	None	N/A	0% EH	0%	0%
	0% Diminished					43% Increased		
						14% HBV		
Non-responders (*N* = 6)	50% Yes	4.6±1.5	33%	67% PPN	55±13	67% EH	17%	33%
	33% Diminished			0% FN		50% Increased		
				17% BN		67% HBV		

*Abbreviations*: PPN, pseudopalisading necrosis; FN, fibrinoid necrosis; BN, background necrosis; EH, endothelial hyperplasia; HBV, hyalinized blood vessels.

**Figure 3:**
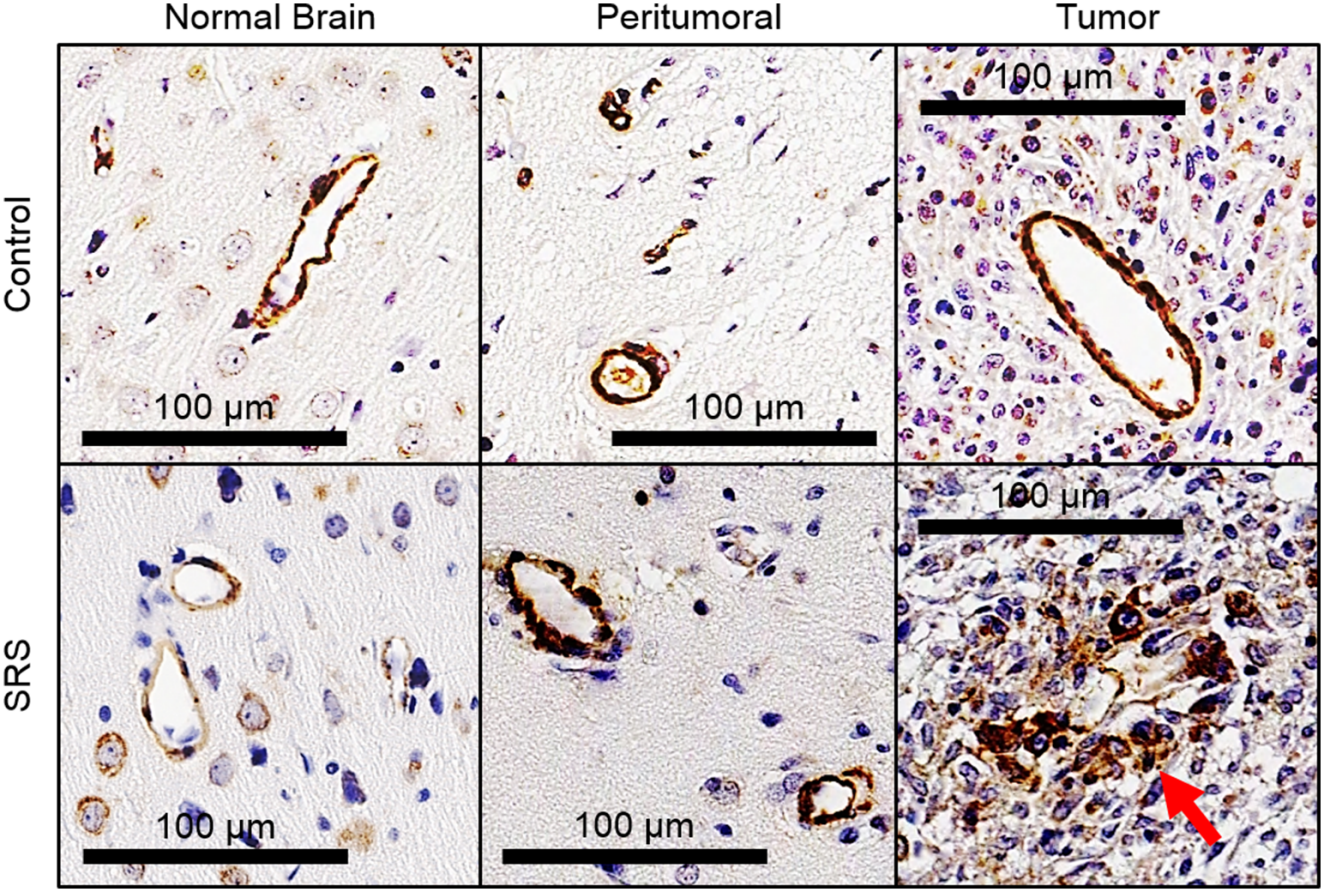
α-smooth muscle actin (α-SMA) positive vessels in the normal brain, peritumoral region, and tumor of a control and a treated animal after stereotactic radiosurgery (SRS). Intact α-SMA positive vessels were observed in control animals, but fragmented coverage of vessels by α-SMA is mostly seen in treated animals (red arrow).

**Figure 4:**
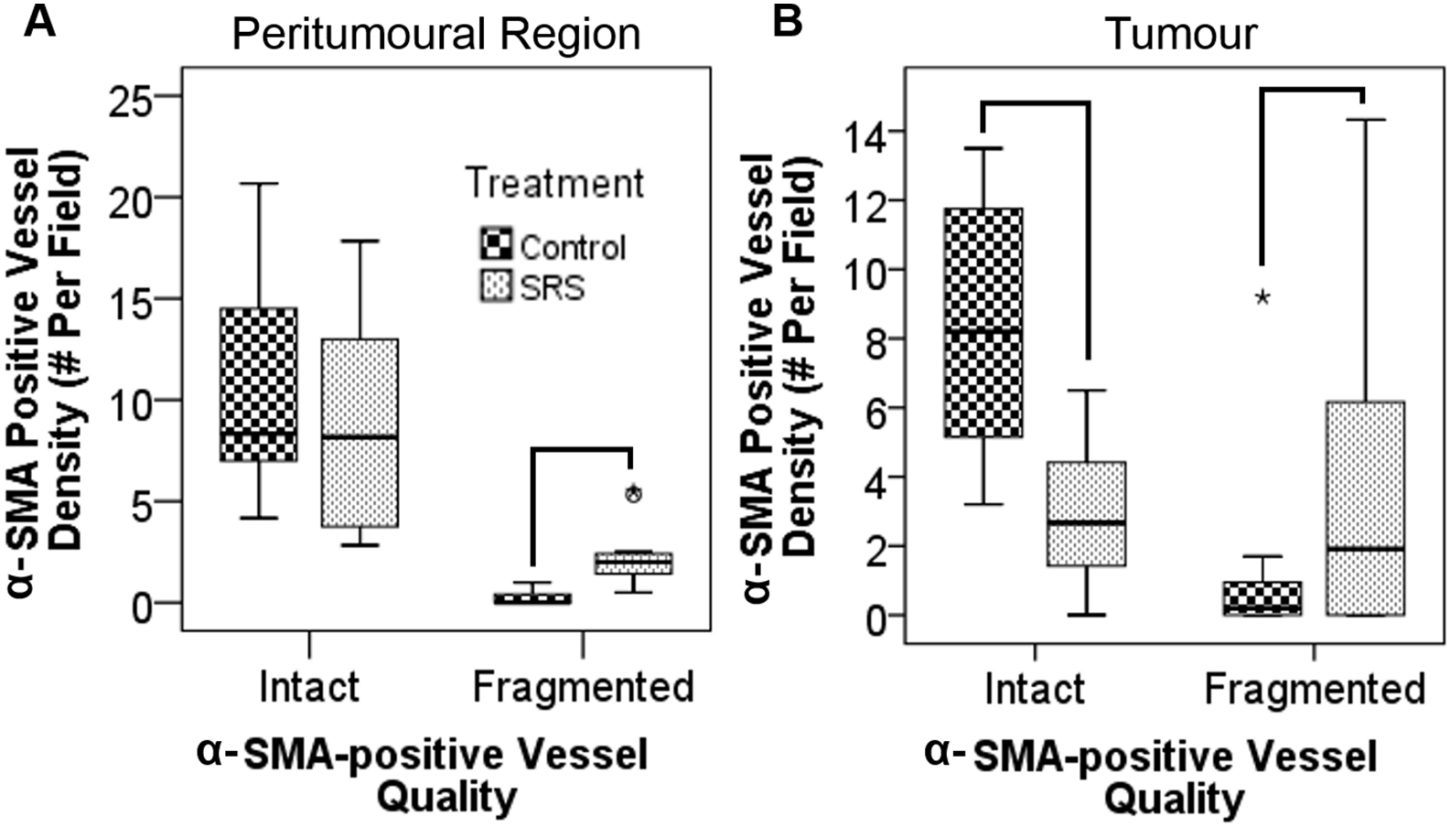
Boxplots of intact and fragmented α-smooth muscle actin (α-SMA) positive vessel densities in (A) peritumoral region and (B) tumor region for the control and SRS groups. Pairs with *P*<0.01 are connected by black lines.

Immunohistochemical staining revealed fragmented α-SMA positive vessels were identified mostly in the SRS-treated tumors (Figure 3). Fragmented α-SMA positive vessel densities in the tumor (*P* = 0.006) and peritumoral regions (*P*<0.001) were significantly higher in the irradiated animals than the control animals (Figure 4). In the tumor, a positive borderline significant correlation between the percentage of fragmented α-SMA positive vessels and PS:BV ratio was identified for the treated animals (*ρ* = 0.58, *P* = 0.06) while a significant negative correlation was found for the control animals (*ρ* = −0.84, *P* = 0.02, correlation graphs not shown). However, the amount of fragmented α-SMA positive vessels in the controls was very small (Figure 4). In the peritumoral region of the treated animals, negative correlations were found between the percentage of fragmented α-SMA positive vessels with rBF (*ρ* = −0.62, *P* = 0.03) and rBV (*ρ* = −0.58, *P* = 0.05).

### Treatment Response, Longitudinal Changes, and Late Histopathologic Features

**Figure 5:**
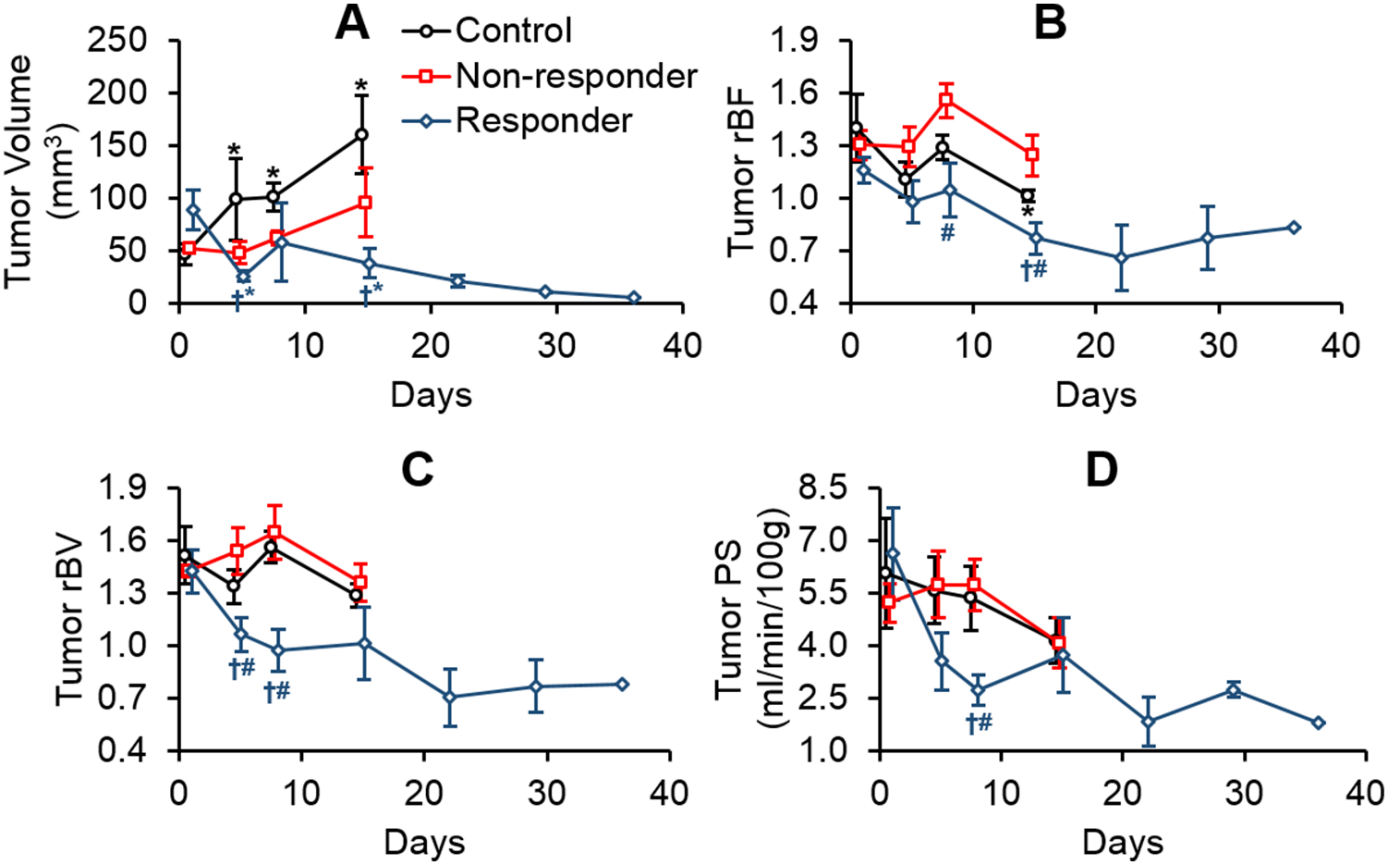
Changes in (A) tumor volume, (B) relative blood flow (rBF), (C) relative blood volume (rBV), and (D) permeability-surface area (PS) in the tumor for controls, responders, and non-responders. *Significantly different from baseline (Friedman test and Wilcoxon-signed rank test). †Significantly different from controls and #significantly different from non-responders (Kruskal-Wallis test followed by Mann-Whitney *U* test).

Median survival of the control group was 10 days post-baseline scan (95% CI = 6–14 days), and median survival of the SRS group was not reached (log-rank *P*<0.04). However, survival in the SRS group was heterogeneous with 46% of the animals not surviving for more than 15 days. We designated these relative low survival animals as non-responders and animals with survival >15 days as responders. Shapiro-Wilk test showed the imaging data were not from a normal distribution (*P*<0.05). Changes in tumor volume, rBF, rBV, and PS of these three groups are shown in Figure 5.

At baseline, there was no statistical difference in tumor volume, rBF, rBV, and PS (*P*>0.15). Similarly, there was no statistical difference in peritumoral rBF, rBV, and PS (*P*>0.37). Significant tumor growth was observed in the controls (Friedman *P* = 0.001, Wilcoxon signed-rank *P*<0.03). Significant tumor shrinkage was observed in the responders (Friedman *P* = 0.04, Wilcoxon signed-rank *P*<0.05). There was no significant tumor volume change in the non-responders. Tumor rBF in the responders was significantly lower than the non-responders and controls on day 14 post-SRS (Kruskal-Wallis *P* = 0.01, Mann-Whitney U *P*<0.04). Tumor rBV in responders were significantly lower than the non-responders and controls on day 4 (Kruskal-Wallis *P* = 0.04, Mann-Whitney U *P*<0.04) and day 7 post-SRS (Kruskal-Wallis *P* = 0.01, Mann-Whitney U *P* = 0.01). Responders’ tumor PS was significantly lower than the other groups on day 7 post-SRS (Kruskal-Wallis *P* = 0.007, Mann-Whitney U *P*≤0.008). Between-group differences and longitudinal changes in PS:BV ratio in the tumor were not significant.

**Figure 6:**
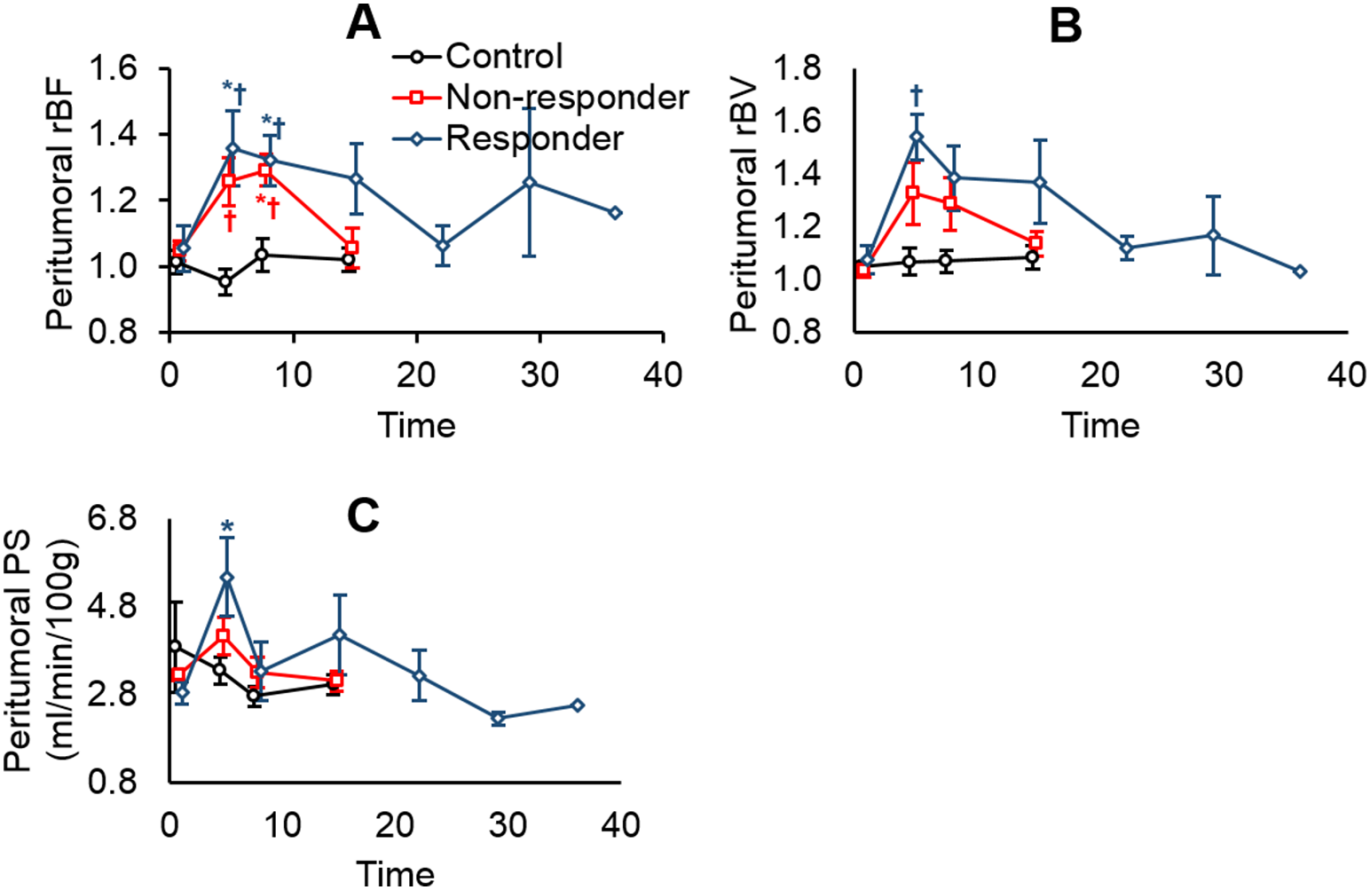
Changes in (A) relative blood flow (rBF), (B) relative blood volume (rBV), and (C) permeability-surface area product (PS) in the peritumoral region for controls, responders, and non-responders. *Significantly different from baseline (Friedman test and Wilcoxon-signed rank test). †Significantly different from controls (Kruskal-Wallis test followed by Mann-Whitney *U* test).

Figure 6 shows an elevation in peritumoral rBF in both responders and non-responders on days 4 and 7 post-SRS compared to the controls (Kruskal-Wallis *P*≤0.006, Mann-Whitney U *P*≤0.008). Significant elevation was observed in peritumoral rBV of responders when compared to controls (Kruskal-Wallis *P*<0.01, Mann-Whitney U *P* = 0.001), and a similar trend was observed for peritumoral PS albeit not significant. This elevation in CT perfusion parameters eventually dissipated with time. Between-group differences and longitudinal changes in PS:BV ratios in the peritumoral region were not significant.

Histological examination revealed no presence of tumor in any of the responders. Increased vascularity was the major sign of late radiation-induced histologic change in the responders (Table 2). Figure 2C shows an example of late radiation-induced damage after the regression of the tumor. For the non-responders, tumors were detected on H&E histology; pseudopalisading necrosis was the major type of necrosis observed. Increased vascularity, endothelial hyperplasia, and hyalinized blood vessels were also observed. The correlations between tumor volume measured from averaged CT and tumor diameter measured from H&E histology were 0.88 (*P* = 0.01) for controls and 0.77 (*P* = 0.05) for non-responders (*P* = 0.01). No tumor was identified on both averaged CT images and H&E histology for the responders.

### Early Prediction of Survival after Stereotactic Radiosurgery

**Figure 7:**
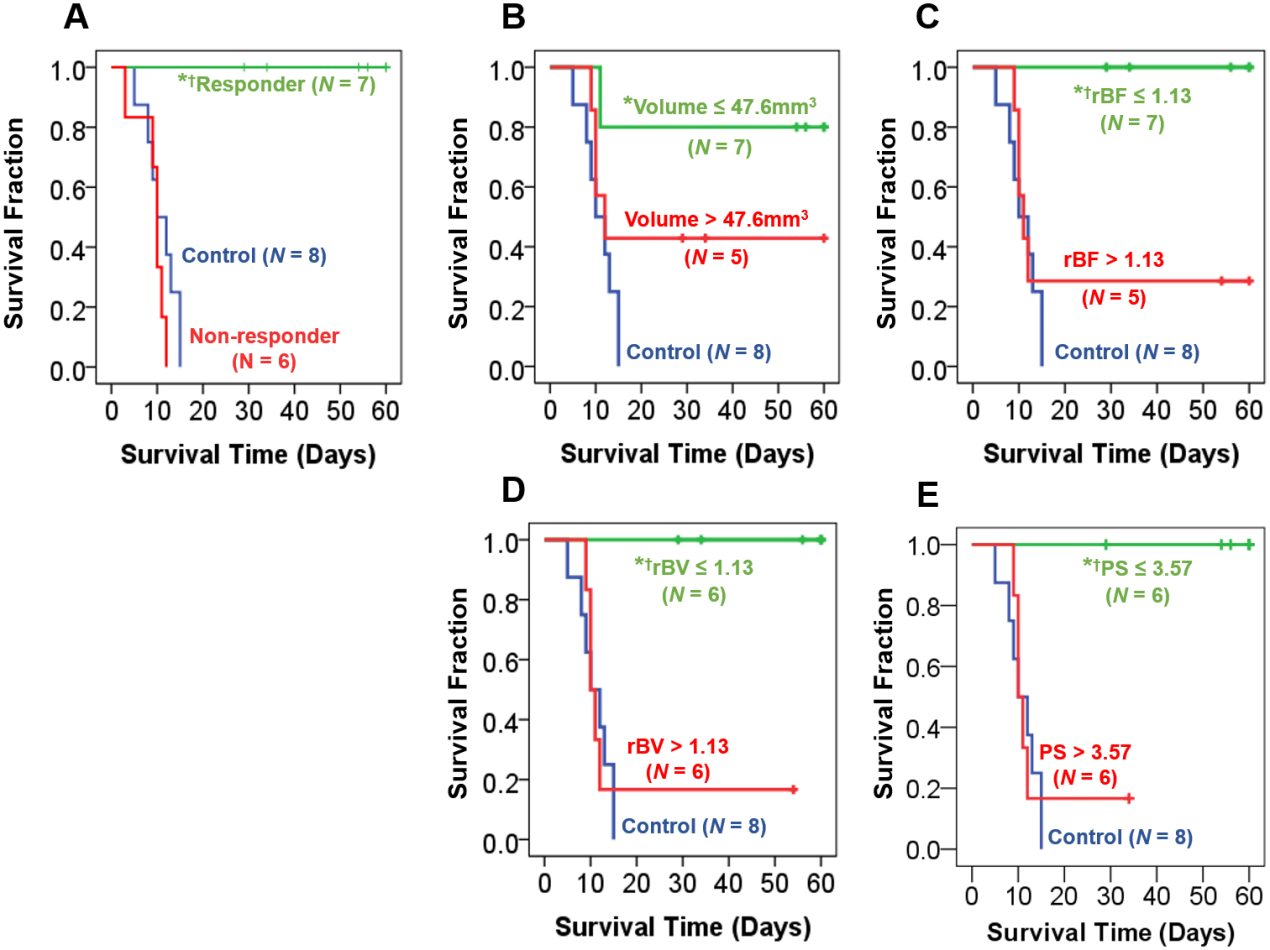
Kaplan-Meier plots of survival categorized by (A) response (B) tumor volume, (C) relative blood flow (rBF), (D) relative blood volume (rBV), and (E) permeability-surface area product (PS). For each imaging parameter, two response groups were identified based on whether the measured value was ≤ lower 50% confidence interval of variations across all treated animals derived from the between-subject variation on day 7 post-SRS. Those that met this criteria were ranked as “low” by applying this threshold, and the others were ranked as “high”. Animals with low rBF, rBV, and PS on day 7 post-SRS showed significantly longer survival than the rest of the treated animals (i.e. high rBF, rBV, and PS) and control animals. *Significantly different from control group. †Significantly different from the other treated animals with a “high” value. Significant at *P*≤0.02 level (log-rank test).

Tumor volume, rBF, rBV, PS, and PS:BV ratio at baseline and on day 4 post-SRS did not correlate with overall survival. However, tumor rBV (*ρ* = −0.61, *P*<0.05) and PS (*ρ* = −0.85, *P* = 0.001) on day 7 post-SRS correlated with overall survival. We evaluated whether each of the imaging parameter was predictive of overall survival by grouping the treated animals based on the measurement on day 7 post-SRS. Kaplan-Meier survival plots are shown in Figure 7. SRS-treated animals with lower tumor rBF, rBV, and PS had significantly longer survival than SRS-treated animals with higher rBF, rBV, and PS (log-rank *P*≤0.02). Treated animals with smaller tumor volumes were associated with longer survival than those with larger tumor volumes, but this was not significant. Similarly, animals with a low PS:BV ratio were not associated with significantly different survival than those with high PS:BV ratio (graph not shown). In terms of predictive performance, both rBV and PS had the highest sensitivity (86%), specificity (100%), and accuracy (92%). rBF had a sensitivity, specificity, and accuracy of 71%, 100%, and 83%, respectively. Tumor volume had a sensitivity, specificity, and accuracy of 57%, 80%, and 67%, respectively. The PS:BV ratio had sensitivity, specificity, and accuracy of 67%, 60%, and 64%, respectively.

## Discussion

Although radiotherapy offers an overall survival benefit at the population level, accurate assessment of tumor response for each patient is crucial for treatment modification if no response to radiation is detected. Therefore, we need to develop non-invasive imaging biomarkers as early indicators of response to radiation.

From preclinical studies, overall survival benefits post-SRS are commonly reported, with some treated animals surviving substantially longer than others [4]–[6]. In our study, SRS demonstrated an overall survival benefit and histological confirmation of radiation-induced damage. This survival benefit is consistent with previously published studies [4]–[6]. Similar to previous studies, we observed substantial heterogeneity in treatment response. We found that CT perfusion can be a potential noninvasive imaging method to predict response to SRS and that rBF, rBV, and PS showed better predictive performance of survival than tumor volume.

In clinical studies, the survival benefit of SRS in combination with fractionated radiotherapy is unclear with some studies suggesting a 2-year overall survival benefit [29], [30], while randomized trials showed that SRS did not confer a survival benefit over fractionated radiotherapy alone [31], [32]. Current clinical evidence from fractionated radiotherapy support our preclinical results in that a higher rBV after the completion of radiotherapy is associated with poor survival [33], [34]. However, the correlation between survival post-SRS and perfusion imaging parameters is lacking in the clinical literature, and there is no imaging biomarker to assess patients who might respond to SRS. Our results provide corroborating evidence to support the hypothesis that CT perfusion is an early biomarker of response to SRS. Use of CT perfusion parameters to characterize tumor vascular profiles and correlate with treatment response might identify patients who would benefit from SRS or hypofractionation treatment schemes. The use of an early imaging biomarker to assess response to SRS or hypofractionated treatment schemes in randomized clinical trials might better define the role of altered fractionation schemes in this group of patients.

From the acute imaging study, PS did not correlate with the percentage of fragmented α-SMA positive vessels, this could because PS is the product between permeability and surface area of the endothelium [18]. Since BV has been shown to correlate with microvessel area [28], we investigated the PS:BV ratio as a surrogate marker of permeability. After SRS, a positive correlation between PS:BV and the percentage of fragmented α-SMA positive vessels points to the effect of radiation on the permeability of the blood-tumor barrier. Vessels that are covered by SMA are mature vessels [24]–[26], and tumor vessel maturity is associated radiation resistance [8], [35]–[36]. From our survival study, it is the permeability-surface area product (PS) that correlated with survival and not the surrogate of permeability (i.e. PS:BV ratio). This suggests a high surface area of SMA coverage after SRS is associated with poor survival. Together, this points to a possible link between the vessel SMA coverage and survival. We were unable to show a correlation between survival and SMA coverage of mature vessels directly since any assessment of tumor vessels would require the sacrifice of animals. Further investigation into the direct associations between tumor PS and SMA coverage of mature vessels with tumor response is warranted.

It is important to study the effect of radiation on the adjacent normal brain tissue (i.e. peritumoral region) because it is usually included in the irradiated volume that receives a significant dose of radiation in clinical practice, particularly for linear accelerator-based SRS. Complications and radiation-induced changes of brain tissue, such as edema and blood-brain barrier breakage, after SRS of arteriovenous malformations correlated with the volume of irradiated tissue that received 12 Gy [37]–[38]. The effect of SRS on peritumoral normal brain region in malignant glioma is not well understood and seldom reported [10]. Zawaski et al. showed that radiation caused significant changes in permeability and leukocyte-endothelial interactions in the peritumoral normal brain, which were indicative of acute inflammatory reaction and radiation-induced astrogliosis. Our study provides supporting evidence by showing an increase in blood-brain barrier PS in the peritumoral region. Therefore, a decrease in tumor rBF, rBV, and PS in the tumor could indicate treatment response, an increase in these parameters in the peritumoral region could be indicative of acute radiation-induced inflammation.

A few limitations of this study must be considered. First, a relationship exists between radiation effect and dose [2], [4]. We chose 12 Gy because this dose can be delivered safely using Helical Tomotherapy, and it is used in the SRS boost of newly diagnosed glioblastoma [29]–[30] and salvage therapy of recurrent glioblastoma for linear accelerator-based SRS [39]. Second, different tumor cell lines display different radiosensitivities [40]. Therefore, dose-response of vascular changes measured by CT perfusion in other glioma models could be different. Thirdly, the sample size was relatively small and all untreated rats and most of the non-responders have died after the fourth CT perfusion scan. Thus, we could not compare the imaging data between these groups at later time points. Longitudinal tumoral and peritumoral changes between the control animals, responders, and non-responders at time points after day 21 post-SRS could be assessed if a larger sample size was available. Finally, while we used stereotactic techniques for radiation of the tumors, treatment volumes were still large relative to the size of the rat brain with the irradiated volume encompassing the ipsilateral cerebral hemisphere. Thus, the volume of normal brain irradiated was larger than would be the case in clinical radiosurgery treatments. It is possible that these volume differences have contributed to the perfusion changes seen in the peritumoral region.

## Conclusions

This study showed that CT perfusion is a candidate to be an early biomarker of response to SRS. A CT perfusion imaging study in the clinical setting would potentially allow for timely and accurate assessment of early response to radiosurgery. It could help understand the role of SRS in these patients and also in combination with anti-angiogenic therapies. Evaluation of CT perfusion imaging in prospective clinical studies are necessary to validate this technique as a predictive assay.

## Supporting Information

Figure S1 **Example of a treatment plan.** The tumor and the 15, 12, and 8 Gy isodose lines are shown in the (A) coronal, (B) axial, and (C) sagittal planes.
(TIF)

Figure S2 **Experiment flowchart.**
(TIF)
