# Supplementary figures and images for: CT Perfusion Imaging as an Early Biomarker of Differential Response to Stereotactic Radiosurgery in C6 Rat Gliomas
Source: PLoS One. 2014 Oct 17;9(10):e109781. doi: 10.1371/journal.pone.0109781 (PMC4201465; doi:10.1371/journal.pone.0109781)

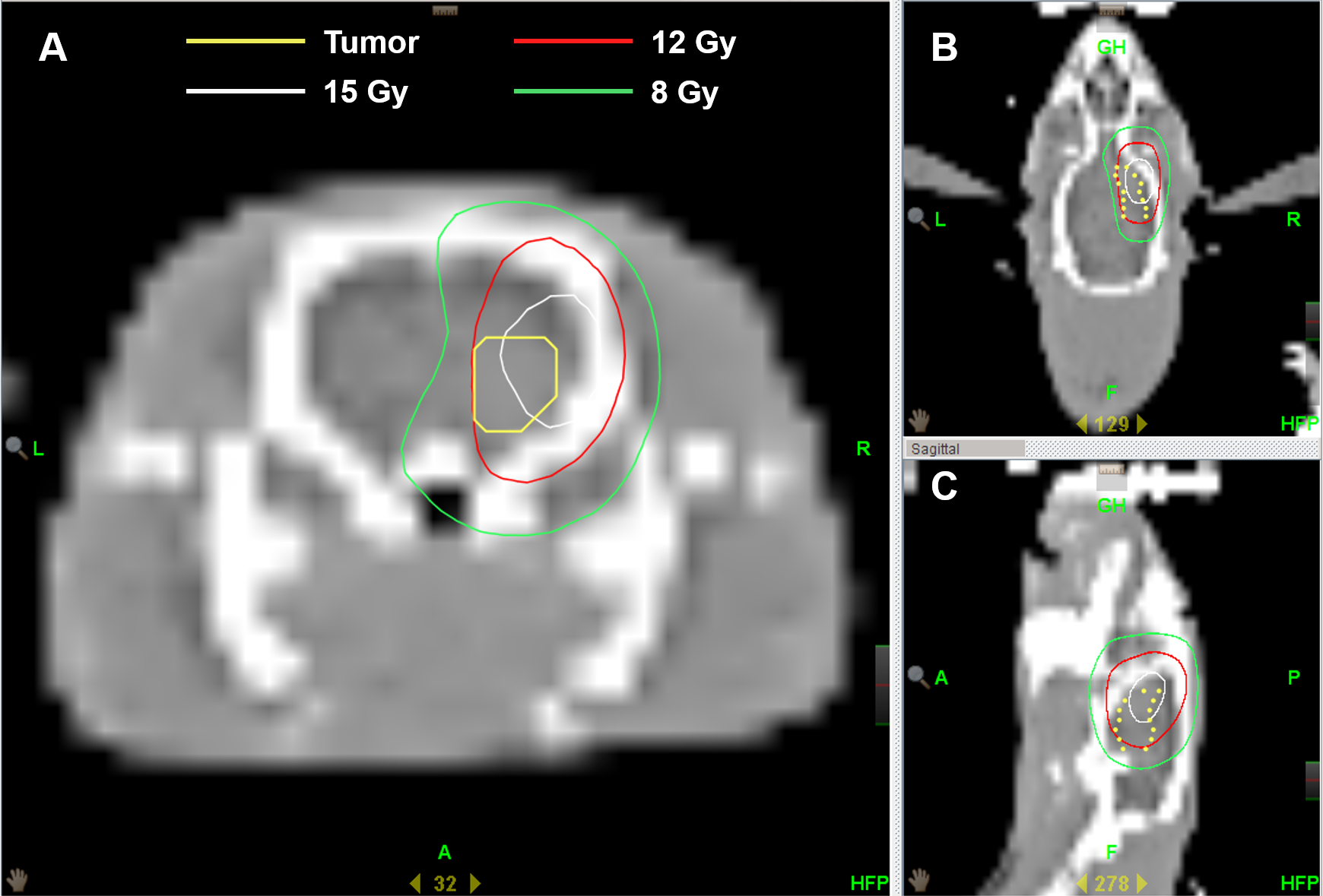

Supplement: Figure S1 — Example of a treatment plan. The tumor and the 15, 12, and 8 Gy isodose lines are shown in the (A) coronal, (B) axial, and (C) sagittal planes. (TIF) [file pone.0109781.s001.tif]

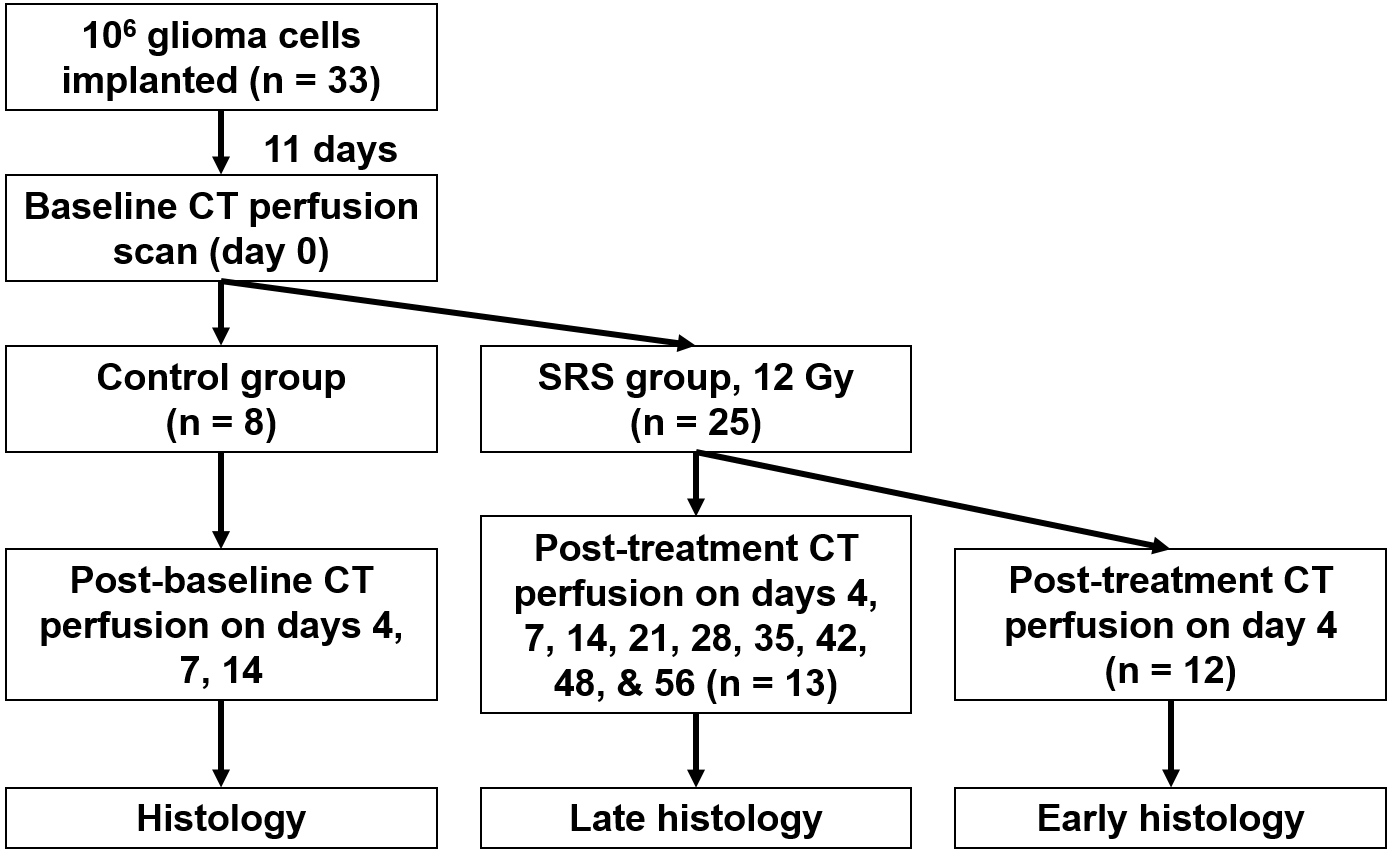

Supplement: Figure S2 — Experiment flowchart. (TIF) [file pone.0109781.s002.tif]
